# Sex Cord Tumor with Annular Tubules: An Incidental Finding in an Endometriotic Cyst—The First Known Cooccurrence

**DOI:** 10.1155/2014/970243

**Published:** 2014-11-02

**Authors:** Meeta Singh, Shramana Mandal, Kaushik Majumdar

**Affiliations:** Department of Pathology, GB Pant Hospital and Associated Maulana Azad Medical College, New Delhi 110002, India

## Abstract

Sex cord tumor with annular tubules (SCTATs) is a relatively rare ovarian neoplasm often having a syndromic association with Peutz-Jeghers syndrome (PJS). Other associations described with this rare neoplasm include adenoma malignum of cervix, Turners syndrome, dysgerminoma, gonadoblastoma, endometrial carcinoma, and endometriosis of fallopian tube. We describe for the first time to the best of our literature search the incidental detection of SCTAT coexisting with an endometriotic cyst of ovary. Meticulous histological scanning and awareness is mandatory for detection of such unusual incidental lesions. Non-PJS SCTATs tend to be larger and could be more prone to distant metastasis, warranting subsequent follow-up.

## 1. Introduction

Sex cord tumor with annular tubules (SCTATs) is a rare ovarian tumor first described in 1970 by Scully [1]. It accounts for only six % of sex cord stromal tumors, which in turn account for only 8% of the overall ovarian neoplasms [1]. Since Scully described it, numerous case reports and a few series have contributed to the better understanding of this rare morphological entity. There is a strong association of ovarian SCTAT and Peutz-Jeghers syndrome (PJS). In a series by Young et al., of 74 cases one third of patients with SCTAT, 27, had PJS [2]. SCTAT has been documented to be an estrogen-progesterone-secreting tumor with low malignant potential. Other associations reported with SCTAT include adenoma malignum of cervix, Turners syndrome, dysgerminoma, gonadoblastoma, endometrial carcinoma, and endometriosis of fallopian tube [2–6]. Present case is the first reported case of two coexisting ovarian pathologies, namely, SCTAT and endometriotic cyst.

## 2. Case History

A 22-year-old lady presented with abdominal pain and abdominal lump for the past one year. On examination a left sided pelvic lump was palpable which was confirmed as ovarian mass on ultrasound and computed tomography (CT scan). On CT scan a well circumscribed multiloculated fluid filled mass was identified measuring 10 × 10 cm with focal solid areas and mural nodules. No lymph nodes or pelvic extension was noted suggestive of benign etiology. Ovarian tumor markers, including carcinoembryonic antigen, carbohydrate antigen 19-9, carbohydrate antigen 125, fetoprotein, and human chorionic gonadotropin, were within the normal range. Serum estradiol levels were within normal limits.

Left oophorectomy was performed, with preoperative assessment of pelvic lymph nodes and peritoneum. Preoperatively, a cystic structure was identified with adhesions to sigmoid and posterior uterine wall. Grossly the mass received was covered by intact pearly white capsule with areas of congestion. On cut section thick and thin walled multiloculated cysts were identified of varying sizes along with solid areas. The cysts were filled with chocolaty fluid. No areas of necrosis/capsular infiltration were identified.

**Figure 1:**
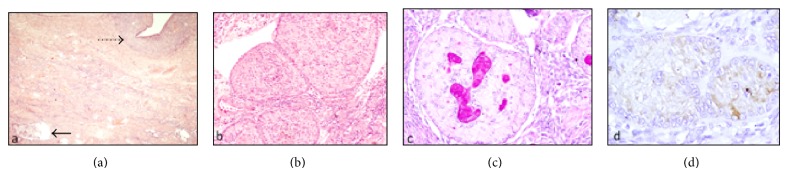
(a) The cysts were lined by endometrial glands and stroma along with numerous hemosiderin laden macrophages (dotted arrow). The solid areas were composed of a cellular tumor having round, complex annular tubules of various sizes (arrow) [HE, 100x]. (b) SCTAT having round, complex annular tubules of various sizes and intercommunicating rings around multiple hyaline bodies [HE, 200x]. (c) Multiple hyaline bodies in the center showing strong PAS positivity [PAS, 200x]. (d) Immunohistochemistry showing tumor cells positive for inhibin [immunohistochemistry with DAB as chromogen, 200x].

On microscopy, the cysts were lined by endometrial glands and stroma along with numerous hemosiderin laden macrophages (Figure 1(a)). The solid areas were composed of a cellular tumor having round, complex annular tubules of various sizes. The tubules were composed of intercommunicating rings around multiple hyaline bodies. The tumor cells were columnar with eosinophilic cytoplasm and round nuclei and exhibited minimal atypia and no mitosis. No capsular infiltration was noted (Figure 1(b)). The cells were bright magenta pink positive for periodic acid schiff (PAS; Figure 1(c)). A possibility of incidental sex cord stromal tumor with annular tubules (SCTATs) was kept and confirmatory immunohistochemistry was performed with CD-10 and inhibin. Both CD-10 and inhibin showed bright cytoplasmic positivity, thus confirming the diagnosis (Figure 1(d)). The contralateral ovary showed only mild oedema with absence of any pathology.

The patient was reexamined and reinvestigated to discard Peutz-Jeghers syndrome. General physical examination and gastrointestinal endoscopy ruled out syndromic SCTAT. Thus a final diagnosis of unilateral sporadic sex cord stromal tumor with annular tubules with coexisting endometriotic cyst was given. No evidence of extraovarian extension/metastasis was noted.

The patient was given only symptomatic and supportive treatment and is well and alive on six months follow-up.

## 3. Discussion

The sex cord tumor with annular tubules (SCTATs) is a distinctive ovarian neoplasm, the predominant component of which has morphologic features intermediate between those of the granulosa cell tumor and those of the Sertoli cell tumor; focal differentiation into either granulosa cell or Sertoli cell tumor may occur. Ultrastructurally, Charcot-Bottcher filament has been noted in some tumors supporting Sertoli cell origin while being absent in some. It was then suggested that SCTAT associated with PJS is a hamartoma while that without is granulosa cell tumor [7].

The majority of patients reported in various series with SCTAT have been in the reproductive age group. Rare pediatric cases have been reported [8]. The clinical manifestations of SCTAT are mainly due to estrogen-progesterone secretion like: menorrhagia, postmenopausal bleeding, precocious puberty, and sterility thus detected usually earlier than ovarian epithelial neoplasms [2, 8–10]. In the present case the patient was in reproductive age group and though did not have any clinical or biochemical features of high estrogen production; he had symptoms related to the endometriotic cyst.

SCTATs have a strong association with PJS and thus can be broadly classified as those with and those without PJS [8]. In the present case no features of PJS were identified. Those associated with PJS tumors are usually benign, multifocal, bilateral, very small, or even microscopic in size and calcified [2]. The ones without PJS are usually unilateral and larger in size [2, 8].

In PJS-associated SCTAT, the serum tumor marker inhibin and immunohistochemical markers inhibin, estrogen receptor, progesterone receptor, and androgen receptor have been studied as diagnostic tools, though morphology is the gold standard [8]. Non-PJS-associated SCTATs also commonly secrete estrogen and progesterone, although the sensitivity is low. In the present case inhibin was strongly positive in the tumor nests along with CD 10 (positive in stromal tumors) positivity; serum estrogen levels were normal in our case.

In addition to its association with PJS, many other coexisting conditions have been reported, mostly with single case reports. These include adenoma malignum of cervix, Turners syndrome, dysgerminoma, gonadoblastoma, endometrial carcinoma, and endometriosis of fallopian tube [2–6]. The latter two conditions can be explained by estrogen production by many of these tumors. Endometriotic cyst coexisting with SCTAT in the same ovary has never been reported in the literature before.

Though malignancy and distant metastasis are known to occur in 20% of non-PJS SCTAT, these tumors are considered to be a tumor with low malignant potential. PJS-associated SCTATs are usually benign [2]. The exact behavior and long term prognosis in these patients are almost unknown and thus the present management guidelines are based on a scanty literature available, which suggests that surgery alone with preservation of fertility (if possible) should be attempted in these patients. A school of thought suggests that management strategies in these patients can be generalized as per the management of granulosa cell tumors [8]. In the present case, only unilateral oophorectomy was performed with examination of peritoneum and pelvic lymph nodes and was apparently well on 6 months follow-up period for after which she was lost to follow-up.

To summarize, a rare case of sporadic SCTAT with endometriotic cyst is presented and its diagnosis and management have been highlighted.
